# S100B Inhibitor Pentamidine Attenuates Reactive Gliosis and Reduces Neuronal Loss in a Mouse Model of Alzheimer's Disease

**DOI:** 10.1155/2015/508342

**Published:** 2015-07-29

**Authors:** Carla Cirillo, Elena Capoccia, Teresa Iuvone, Rosario Cuomo, Giovanni Sarnelli, Luca Steardo, Giuseppe Esposito

**Affiliations:** ^1^Laboratory for Enteric NeuroScience (LENS), Translational Research Center for Gastrointestinal Disorders (TARGID), University of Leuven, 3000 Leuven, Belgium; ^2^Department of Physiology and Pharmacology “Vittorio Erspamer”, La Sapienza University of Rome, 00185 Rome, Italy; ^3^Department of Pharmacy, Federico II University of Naples, 80131 Naples, Italy; ^4^Department of Clinical Medicine and Surgery, Federico II University of Naples, 80131 Naples, Italy

## Abstract

Among the different signaling molecules released during reactive gliosis occurring in Alzheimer's disease (AD), the astrocyte-derived S100B protein plays a key role in neuroinflammation, one of the hallmarks of the disease. The use of pharmacological tools targeting S100B may be crucial to embank its effects and some of the pathological features of AD. The antiprotozoal drug pentamidine is a good candidate since it directly blocks S100B activity by inhibiting its interaction with the tumor suppressor p53. We used a mouse model of amyloid beta- (A*β*-) induced AD, which is characterized by reactive gliosis and neuroinflammation in the brain, and we evaluated the effect of pentamidine on the main S100B-mediated events. Pentamidine caused the reduction of glial fibrillary acidic protein, S100B, and RAGE protein expression, which are signs of reactive gliosis, and induced p53 expression in astrocytes. Pentamidine also reduced the expression of proinflammatory mediators and markers, thus reducing neuroinflammation in AD brain. In parallel, we observed a significant neuroprotection exerted by pentamidine on CA1 pyramidal neurons. We demonstrated that pentamidine inhibits A*β*-induced gliosis and neuroinflammation in an animal model of AD, thus playing a role in slowing down the course of the disease.

## 1. Introduction

Alzheimer's disease (AD) is the most common age-related neurodegenerative disorder [1], whose pathologic hallmarks are the deposit of neurofibrillary tangles and senile plaques (beta-amyloid protein deposits) in the brain [2, 3]. Increasing evidence demonstrates that inflammation in the brain, specifically neuroinflammation, plays a key role in the development of AD [4, 5]. This pathologic event is accompanied by the activation of glial cells in the brain, a phenomenon known as “reactive gliosis” [6]. In fact, it has been shown that amyloid-beta- (A*β*-) induced reactive gliosis and the consequent inflammatory responses with the release of neurotoxic cytokines are present in the AD brain and prominently contribute to the progression of the disease [7]. The two events are thus thoroughly linked and are object of the current research on AD pathophysiology.

The definition of “reactive gliosis” refers to the overexpression of glial-derived factors. Amongst all, one of the most interesting from a pharmacological point of view is the protein S100B [8, 9]. This small and soluble protein, belonging to the large family of EF-related Ca^++^- and Zn^++^-binding proteins, plays a dual effect. While at nanomolar concentrations S100B provides to a prosurvival effect on neurons and stimulates neurite outgrowth, at higher (micromolar) concentrations it promotes inflammation and neuronal apoptosis [10]. S100B overexpression has been linked to the typical features of reactive gliosis in AD [11, 12]. After release, and only when it reaches micromolar concentrations, the protein accumulates at the RAGE (receptor for advanced glycation end-products) surface [13–15]. Such interaction leads to the phosphorylation of mitogen-activated protein kinase (MAPK) and the activation of nuclear factor-kappaB (NF-*κ*B). This cascade, in turn, promotes the transcription of proinflammatory cytokines and inducible nitric oxide synthase (iNOS) protein [16]. The possibility of interfering with this harmful cycle, by directly targeting S100B, might therefore represent a novel approach to embank neurotoxicity in AD brain.

Pentamidine isethionate, discovered in 1938 as an antiprotozoal drug and approved in the United States for the treatment of* Pneumocystis carinii* pneumonia and other protozoal diseases [17], appears to be an intriguing candidate. In fact, in addition to the antiprotozoal activity, pentamidine also inhibits S100B-mediated effects because of its ability to block S100B/p53 interaction [18]. In spite of the several data showing the anti-inflammatory effect exerted by pentamidine due to S100B inhibition [19–22], no data on the possible effect of pentamidine on gliosis and neuroinflammation in AD models are available so far.

Based on this background, the present study was aimed at evaluating the effect of a daily intrahippocampal administration of pentamidine in a mouse model of AD characterized by A*β*-induced gliosis and neuroinflammation. Because of the capability to inhibit S100B protein, we investigated (1) the effect exerted by pentamidine on reactive gliosis, (2) the molecular mechanism by which pentamidine might interfere with reactive gliosis, and (3) whether pentamidine-mediated inhibition of reactive gliosis may result in the rescue of neuronal loss in AD brain.

## 2. Methods

### 2.1. Ethics Statement

All the experiments were performed in accordance with the National Institutes of Health Guidelines for the Care and Use of Laboratory Animals (Institute of Laboratory Animal Resources, 1996) and those of the Italian Ministry of Health (D.L. 116/92). The study was approved by the Institutional local Animal Care and Use Committees.

### 2.2. Animals

Experiments were performed in C57BL/6J mice (3–5 months old, weight range: 35–40 g; Harlan, Udine, Italy). Animals were housed under controlled illumination (12 h light/12 h dark cycle) and standard environmental conditions (room temperature 20–22°C, humidity 55–60%). Food and water were available* ad libitum*. All surgery and experimental procedures were performed during the light cycle. All efforts were made to reduce the number of animals used and the suffering during surgical experiments.

### 2.3. Surgical Preparation and Intrahippocampal Injection

Mice (total *n* = 40) were anesthetized i.p. with pentobarbital (40 mg/kg). They were then placed in a stereotaxic frame and injected in the hippocampi (CA1 area) with human A*β* (1–42) peptide (Tocris Cookson, UK). The coordinates for the injection were −1.58 mm posterior from bregma, ±1.2 mm lateral and 1.60 mm ventral to the skull surface. A*β* peptide was dissolved in ALZET artificial cerebrospinal fluid according to the manufacturer's instructions (ALZET-company, Cupertino, CA, USA). The final concentration was 10 *μ*g/mL and a volume of 3 *μ*L was injected using an ALZET microdialysis pump by keeping the flow at the constant speed of 0.5 mL/min. Control mice (vehicle-treated group, *n* = 8) were injected with an equivalent volume of artificial cerebrospinal fluid. Starting at the third day after surgery and using the previously implanted cannula, three groups of mice (*n* = 8 per group) received intrahippocampal infusion of pentamidine (0.05–5 *μ*g/mL/day) for consecutive 7 days. At the end of treatments, the cannula was removed and the animals, to prevent damage to the scalp sutures, were kept in individual cages until they were killed for tissue processing.

### 2.4. Immunohistochemistry and Immunofluorescence Analyses

Immunohistochemistry analysis was performed on hippocampal coronal sections (adjacent to the site of the injection) obtained from the brains of vehicle-, A*β*-, and pentamidine-treated mice. Sections were incubated for 2 hours with blocking buffer (PBS containing 15 mM NaN_3_, 10% albumin, and 0.25% Triton X-100) and then with mouse anti-GFAP antibody (1 : 400, Sigma-Aldrich, Milan, Italy) overnight at 4°C. Biotinylated secondary antibody (1 : 200; Vector Laboratories, Peterborough, UK) and the preformed avidin biotinylated peroxidase complex (VECTASTAIN ABC kit; Vector Laboratories) were then added and the reaction was revealed by 3,3-diaminobenzidine tetrahydrochloride (Sigma-Aldrich). Representative pictures were captured using a high-resolution digital camera (Nikon Digital Sight DS-U1).

For the immunofluorescence, hippocampal coronal sections obtained from the brains of vehicle-, A*β*-, and pentamidine-treated mice were blocked in 10% albumin bovine serum 0.1% Triton-PBS solution for 90 min and subsequently exposed for 1 h to rabbit anti-GFAP antibody (1 : 1000, Abcam, Cambridge, UK) and mouse anti-p53 antibody (1 : 500, Abcam). Sections were then incubated in the dark with the proper secondary antibody: fluorescein isothiocyanate-conjugated anti-rabbit (1 : 1000, Abcam) or Texas Red-conjugated anti-mouse (1 : 1000, Abcam), respectively. Immunofluorescence was analyzed with a Nikon Eclipse 80i microscope (Nikon Instruments Europe, Kingston upon Thames, UK) and images were captured by a high-resolution digital camera (Nikon Digital Sight DS-U1). The number of GFAP+ or p53+ cells was then calculated in every tenth coronal section spanning the ipsilateral hippocampus at the injection site using unbiased stereology (Stereo Investigator, MBF, Williston, VT, USA). According to the manufacturer's protocol, a counting frame (15 × 15 × 20 *μ*m) was placed at the intersection of a matrix (200 × 200 *μ*m) randomly superimposed by the software onto the region of interest.

### 2.5. Nissl Staining

Hippocampal coronal sections (*n* = 5) obtained from the brains of vehicle-, A*β*-, and pentamidine-treated mice were sequentially dipped in different alcohol solutions of decreasing percentage to remove lipids from the tissue, stained with 2% cresyl violet solution for 5 minutes, and dehydrated with a series of baths with increasing alcohol percentage solutions. Sections were analyzed by a blind observer through a Nikon Eclipse 80i microscope. Representative pictures were captured using a high-resolution digital camera (Nikon Digital Sight DS-U1) and analyzed using NIS-Elements software (Nikon Instruments Europe). The extent of neuronal damage was expressed as the ratio between the number of nonstained (death) neurons and the total number of neurons per mm of length of CA1 area in injected ipsilateral hippocampi, according with the following formula:

(1) death  neurons  per  CA1  mm2  areatotal  neurons  per  CA1 mm2  area =extent  of  CA1  damage  %.

### 2.6. Fluoro-Jade B Staining

To further evaluate neuronal loss/rescue in the hippocampus, Fluoro-Jade B (FJB) staining was performed on hippocampal coronal sections obtained from the brains of vehicle-, A*β*-, and pentamidine-treated mice. Sections were immersed in a basic alcoholic solution for 6 minutes and 0.06% KMnO_4_ for 15 minutes. Sections were then incubated in 0.0004% FJB (Histo-Chem, Jefferson, AR, USA) for 20 minutes, washed in distilled water, and then dried. To quantify neuronal death, every tenth coronal section spanning hippocampus was analyzed from each animal (*n* = 5). A blinded observer counted the number of FJB-positive neurons in the hippocampal CA1 from ipsilateral hemispheres to the injection site. Mean counts of FJB-positive neurons from each region were used for the statistical analysis.

### 2.7. Immunoblot Analysis

Ipsilateral hippocampi to the injection site were dissected from frozen excised brains of vehicle-, A*β*-, and pentamidine-treated mice and lysed with ice-cold hypotonic lysis buffer (Tris/HCl pH 7.5 50 mM; NaCl 150 mM; EDTA 1 mM; Triton X-100 1%) supplemented with the proper protease inhibitor cocktail (Roche, Milan, Italy) and incubated on ice for additional 15 min. Total protein extracts were obtained by centrifugation at 13,000 g for 15 min at 4°C. Samples were subjected to SDS-polyacrylamide gel electrophoresis and proteins were transferred onto nitrocellulose membrane and incubated with one of the following antibodies: anti-GFAP (1 : 50000); anti-iNOS (1 : 200); anti-COX-2 (1 : 1000), anti-phospho(p)-p38 MAPK (1 : 400), anti-RAGE (1 : 1000), and anti-*β*-actin (1 : 2000) (all from Abcam). After wash in TBS 1X with 0.1% Tween 20, the membrane was incubated for 2 h at room temperature with the appropriate secondary HRP-conjugated antibodies anti-mouse (1 : 1000, Abcam) or anti-rabbit (1 : 1000, Abcam). Lastly, the membrane was exposed to the enhanced chemiluminescence substrate (ECL, Invitrogen, Milan, Italy), the immunoreactive bands were revealed through a Versadoc (Bio-Rad Laboratories, Milan, Italy) and the digital images were analyzed with the Quantity One Software (Bio-Rad Laboratories).

### 2.8. Electrophoretic Mobility Shift Assay (EMSA)

EMSA was performed to detect NF-*κ*B activation in hippocampal homogenates obtained from the brains of vehicle-, A*β*-, and pentamidine-treated mice. Double stranded oligonucleotides containing NF-*κ*B recognition sequence (5′AGTTGAGGGGACTTTCCCAGGC-3′) were end-labeled with ^32^P-*γ*-ATP (Amersham, Milan, Italy). Nuclear extracts were incubated for 15 min with radiolabeled oligonucleotides (2.5–5.0 × 10^4^ cpm) in 20 mL reaction buffer containing 2 mg poly dI-dC (Boehringer-Mannheim, Milan, Italy), 10 mM Tris–HCl (pH 7.5), 100 mM NaCl, 1 mM EDTA, 1 mM dl-dithiothreitol, 1 mg/mL bovine serum albumin, and 10% glycerol. Nuclear protein-oligonucleotide complexes were resolved by electrophoresis on a 6% nondenaturing polyacrylamide gel in 1X Tris Borate EDTA buffer at 150 V for 2 hrs at 4°C. The gel was dried and autoradiographed with an intensifying screen at −80°C for 20 h. Subsequently, the relative bands were quantified by densitometric scanning with Versadoc (Bio-Rad Laboratories) and computer software (Quantity One Software, Bio-Rad Laboratories). Oligonucleotide synthesis was performed to our specifications by Tib Molbiol (Boehringer-Mannheim, Ingelheim am Rhein, Germany).

### 2.9. Nitrite Assay

NO was measured as nitrite (NaNO_2_) accumulation in mice hippocampal homogenates, obtained from the brains of vehicle-, A*β*-, and pentamidine-treated mice, by using the Griess method [23]. Briefly, Griess reagent (1% sulphanilamide, 0.1% naphthylethylenediamine in H_3_PO_4_) was added to an equal volume of tissue homogenate and the absorbance of the reaction product was measured at 550 nm. Nitrite concentration (nM) was thus determined using a standard curve of NaNO_2_.

### 2.10. Lipid Peroxidation Assay

Malonyl dialdehyde (MDA) was measured by the thiobarbituric acid colorimetric assay in mice hippocampal homogenates obtained from the brains of vehicle-, A*β*-, and pentamidine-treated mice. Briefly, 1 mL trichloroacetic acid 10% was added to 450 *μ*L of tissue lysate. After centrifugation, 1.3 mL thiobarbituric acid 0.5% was added and the mixture was heated at 80°C for 20 min. After cooling, MDA formation was recorded (absorbance 530 nm and absorbance 550 nm) in a PerkinElmer (Waltham, MA, USA) spectrofluorometer and the results were presented as ng MDA/mL.

### 2.11. Enzyme-Linked Immunosorbent Assay (ELISA) for Prostaglandin (PGE)2, S100B, and Interleukin- (IL-) 1beta (**β**)

ELISA for PGE2, IL-1*β*, and S100B (all from R&D Systems, Minneapolis, Minnesota, USA) was carried out on mouse hippocampal homogenates, obtained from the brains of vehicle-, A*β*-, and pentamidine-treated mice, according to the manufacturer's protocol. Absorbance was measured on a microtiter plate reader. PGE2, IL-1*β*, and S100B levels were determined using standard curves method.

### 2.12. Statistical Analysis

Results were expressed as mean ± SEM of *n* experiments. Statistical analysis was performed using analysis of variance (ANOVA) and multiple comparisons were performed by Bonferroni's test, with *P* < 0.05 considered as significant.

## 3. Results 

### 3.1. Pentamidine Attenuates A**β**-Induced Gliosis and Neuroinflammation in Hippocampi

**Figure 1:**
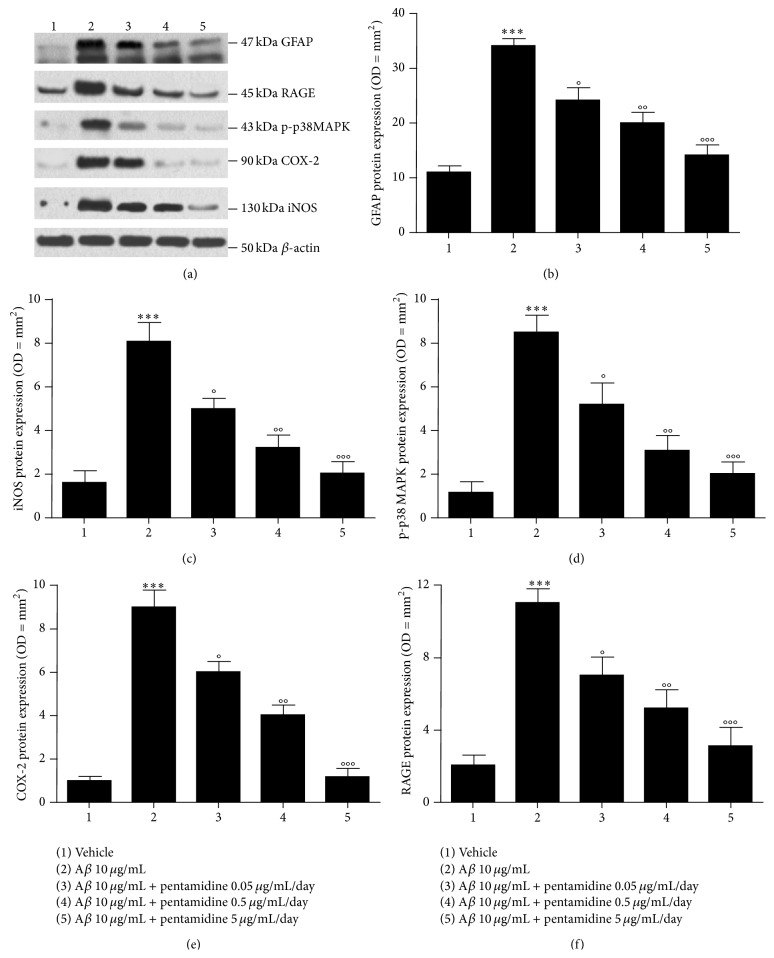
(a) Western blot and (b–f) densitometric analysis (arbitrary units normalized on the expression of the housekeeping protein *β*-actin) showing the effect of 7 days of intrahippocampal injection of pentamidine (0.05–5 *μ*g/mL/day) on GFAP (b), iNOS (c), p-p38 MAPK (d), COX-2 (e), and RAGE (f) expression in A*β*-injected mice. Results are expressed as mean ± SEM of *n* = 5 experiments performed in triplicate. ^*∗∗∗*^ *P* < 0.001 versus vehicle-treated mice; °*P* < 0.05, °°*P* < 0.01 and °°°*P* < 0.001 versus A*β*-treated mice.

Immunoblot analysis showed that A*β* injection significantly increased the expression of GFAP (34.0 ± 1.6 versus 11.0 ± 1.2, *P* < 0.001, Figures 1(a) and 1(b)), iNOS (8.1 ± 0.9 versus 1.6 ± 0.6, *P* < 0.001, Figures 1(a) and 1(c)), p-p38 MAP-kinase (8.6 ± 0.8 versus 1.2 ± 0.5, *P* < 0.001, Figures 1(a) and 1(d)), and COX-2 (9.0 ± 0.8 versus 1.0 ± 0.2, *P* < 0.001, Figures 1(a) and 1(e)) proteins in hippocampal homogenates, compared to vehicle-treated mice. In the same way, also extracellular RAGE protein expression was significantly increased (11.0 ± 0.8 versus 2.1 ± 0.6, *P* < 0.001, Figures 1(a) and 1(f)) in the hippocampi of A*β*-injected compared to vehicle-treated mice. Treatment with pentamidine (0.05–5 *μ*g/mL/day) for 7 days, markedly and in dose-dependent manner blunted A*β*-induced overexpression of GFAP (24.0 ± 2.3, 20.0 ± 2.0 and 14.0 ± 2.0 versus 34.0.3 ± 1.6, *P* < 0.05, 0.01 and 0.001, resp., Figures 1(a) and 1(b)), iNOS (5.0 ± 0.5, 3.2 ± 0.3 and 2.0 ± 0.6 versus 8.1 ± 0.9, *P* < 0.05, 0.01 and 0.001, resp., Figures 1(a) and 1(c)), p-p38 MAPK (5.2 ± 1.0, 3.1 ± 0.7 and 2.0 ± 0.6 versus 8.5 ± 0.8, *P* < 0.05, 0.01 and 0.001, resp., Figures 1(a) and 1(d)), and COX-2 (6.0 ± 0.5, 4.0 ± 0.5 and 1.2 ± 0.4 versus 9.0 ± 0.8, *P* < 0.05, 0.01 and 0.001, resp., Figures 1(a) and 1(e)) in hippocampi homogenates, compared to A*β*-treated mice. The expression of the extracellular RAGE was also significantly and concentration-dependent reduced by pentamidine treatment (0.05–5 microg/mL/day) for 7 days (7.0 ± 1.0, 5.2 ± 1.0 and 3.1 ± 1.0 versus 11.0 ± 0.4, *P* < 0.05, 0.01 and 0.001, resp., Figures 1(a) and 1(f)).

**Figure 2:**
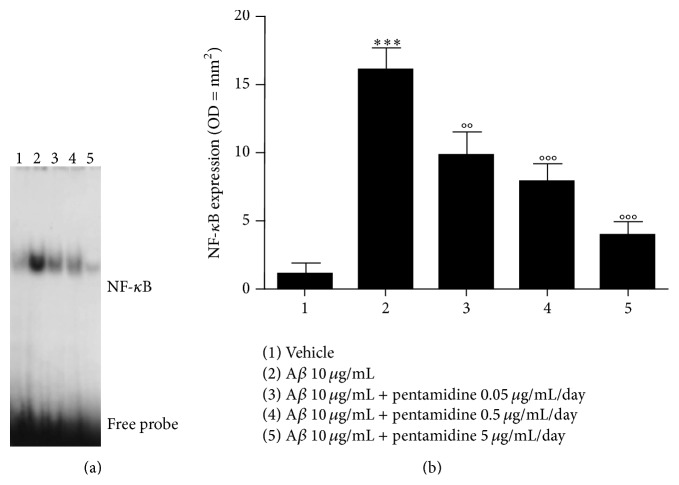
(a) Electrophoretic mobility shift assay (EMSA) and the relative (b) densitometric analysis showing the effect following 7 days of intrahippocampal injection of pentamidine (0.05–5 *μ*g/mL/day) on the expression of NF-*κ*B in A*β*-injected mice. Results are expressed as mean ± SEM of *n* = 5 experiments performed in triplicate. ^*∗∗∗*^ *P* < 0.001 versus vehicle-treated mice; °°*P* < 0.01 and °°°*P* < 0.001 versus A*β*-treated mice.

At nuclear level, A*β* injection induced a significant upregulation of NF-*κ*B (17.5 ± 1.9 versus 1.3 ± 0.8, *P* < 0.001, Figures 2(a) and 2(b)) compared to vehicle-treated mice, as demonstrated by EMSA analysis, indicating a marked A*β*-induced neuroinflammatory response in the hippocampi (Figures 2(a) and 2(b)). Pentamidine-mediated inhibitory effect was observed also for NF-*κ*B in the nuclear extracts, which was significantly and dose dependently downregulated (10.6 ± 2.0, 8.4 ± 1.5 and 4.4 ± 1.4 versus 17.5 ± 1.9, *P* < 0.05, 0.01 and 0.001, resp., Figures 2(a) and 2(b)).

**Figure 3:**
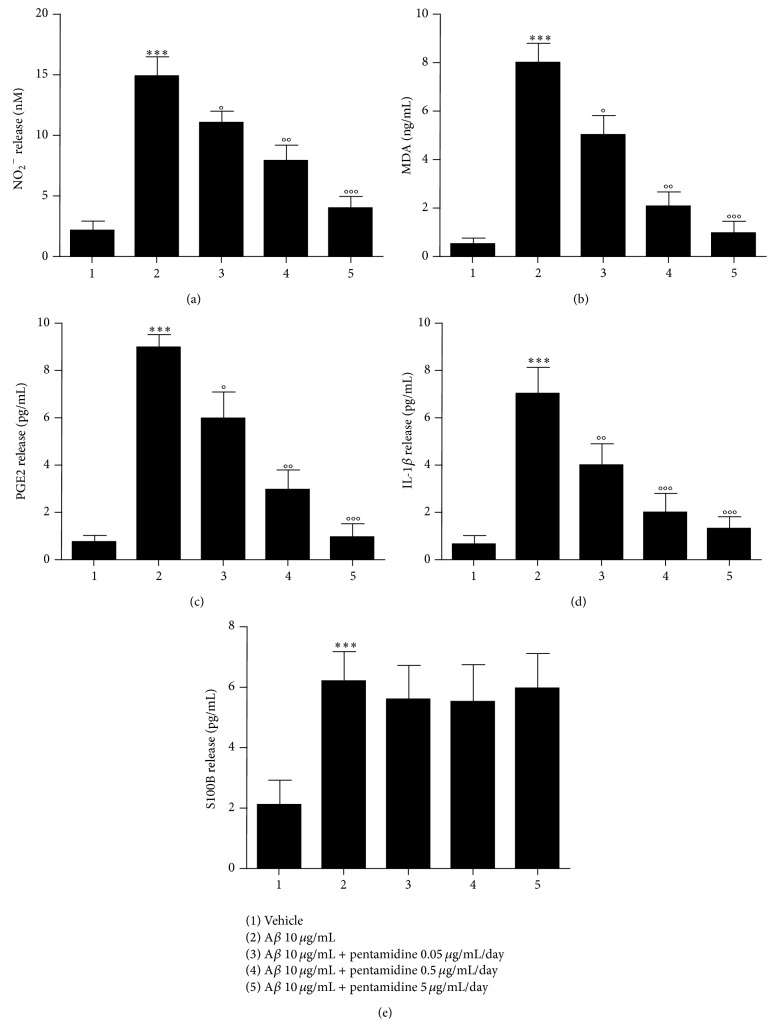
Effect of pentamidine on release of nitrites (a), MDA (b), PGE2 (c), IL-1*β* (d), and S100B (e) in hippocampal homogenates of A*β*-injected mice. Results are expressed as mean ± SEM of *n* = 5 experiments performed in triplicate. ^*∗∗∗*^ *P* < 0.001 versus vehicle-treated mice; °*P* < 0.05, °°*P* < 0.01 and °°°*P* < 0.001 versus A*β*-treated mice.

As expected, lipid peroxidation assay and Griess reaction showed that A*β* injection area caused a significant increase of nitrite (15.6 ± 1.9 versus 2.0 ± 0.8, *P* < 0.001, Figure 3(a)) and MDA (8.0 ± 0.8 versus 0.6 ± 0.1, *P* < 0.001, Figure 3(b)) in the hippocampi of A*β*-treated mice, as a sign of ongoing inflammation. ELISA also showed that PGE2 (8.7 ± 0.4 versus 0.7 ± 0.1, *P* < 0.001, Figure 3(c)), IL-1*β* (6.8 ± 1.0 versus 0.7 ± 0.1, *P* < 0.001, Figure 3(d)), and S100B (6.1 ± 1.2 versus 2.0 ± 1.0, *P* < 0.001, Figure 3(e)) released in the hippocampi of A*β*-injected mice were significantly increased compared to vehicle-treated mice. In line with immunoblot analysis, pentamidine treatment (0.05–5 *μ*g/mL/day) for 7 days caused a marked and dose-dependent attenuation of all the abovementioned proinflammatory markers in the hippocampi: nitrite (11.3 ± 1.4, 8.1 ± 1.5 and 4.4 ± 1.4, *P* < 0.05, 0.01 and 0.001, resp., Figure 3(a)); MDA (4.7 ± 0.8, 2.0 ± 0.5 and 0.9 ± 0.3 versus 8.0 ± 0.8, *P* < 0.05, 0.01 and 0.001, resp., Figure 3(b)); PGE2 (6.0 ± 1.0, 2.8 ± 0.8 and 1.0 ± 0.4, *P* < 0.05, 0.01 and 0.001, resp., Figure 3(c)); and IL-1*β* (3.9 ± 0.8, 1.9 ± 0.8 and 1.2 ± 0.4, *P* < 0.01 and 0.001, resp., Figure 3(d)) compared to A*β*-treated mice. Only the release of S100B remained unaffected (5.8 ± 1.3, 5.8 ± 1.3 and 5.9 ± 1.2, resp., all *P* > 0.05, Figure 3(e)) in pentamidine-treated compared to A*β*-treated mice.

### 3.2. Pentamidine Inhibits Reactive Gliosis, Reduces Astrocyte Infiltration, and Rescues Neuronal Loss in A**β**-Injected Mice

**Figure 4:**
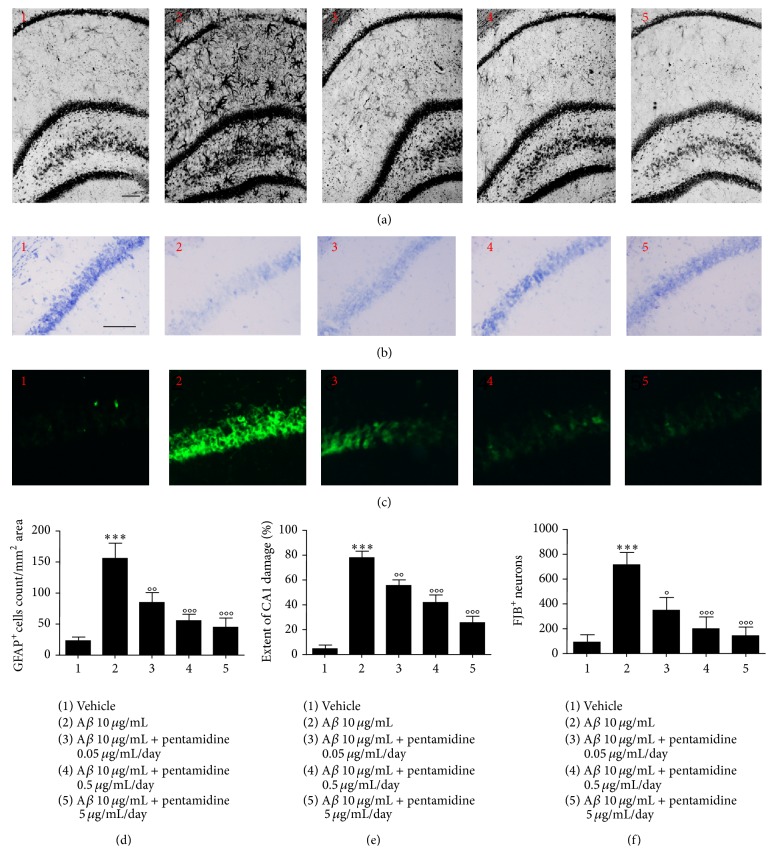
(a) Immunohistochemistry analysis showing the effect of pentamidine in hippocampal coronal sections after A*β* injection. The upper panel shows GFAP-positive cells (astrocytes) infiltrating the hippocampi. Note the increased number of GFAP-positive cells in A*β*-treated (2) compared to vehicle-treated mice (1) and the dose-dependent reduction after pentamidine treatment (3-4-5). Scale bar: 200 *μ*m. (b) Nissl staining showing the effect of pentamidine on pyramidal neuron loss in the CA1 area after A*β* injection. Note the reduced number of neurons stained in A*β*-treated (2) compared to vehicle-treated mice (1) and the dose-dependent reduction of neuronal loss after pentamidine treatment (3-4-5). Scale bar: 200 *μ*m. (c) Immunofluorescence analysis showing the effect of pentamidine in hippocampal coronal sections after A*β* injection. Note the reduced number of neurons after A*β* injection (2) compared to vehicle-treated mice (1) and the dose-dependent neuroprotection after pentamidine treatment (3-4-5). Scale bar: 200 *μ*m. (d) Relative quantification of GFAP expression, (e) extent of CA1 damage measurement, and (f) number of neurons stained with Fluorojade B (FJB) in the hippocampi. Results are expressed as mean ± SEM of *n* = 5 experiments performed in triplicate. ^*∗∗∗*^ *P* < 0.001 versus vehicle-treated mice; °°*P* < 0.01 and °°°*P* < 0.001 versus A*β*-treated mice.

A*β* injection caused a marked glia activation, as shown by the increased expression of the astrocytic marker GFAP (153 ± 25 versus 23 ± 5.8, *P* < 0.001, Figures 4(a) and 4(d)). In parallel, Nissl staining indicated that A*β* injection caused a severe neuronal loss, especially in the CA1 area (site of injection), compared to vehicle-treated mice (79 ± 5.0 versus 5.6 ± 2, *P* < 0.001, Figures 4(b) and 4(e)). Treatment with pentamidine (0.05–5 *μ*g/mL/day) for 7 days concentration dependently rescued neurons integrity in the CA1 area (58 ± 4.1, 42 ± 7.0 and 22 ± 5.0 versus 79 ± 5.0, *P* < 0.01 and 0.001, resp., Figures 4(b) and 4(e)).

The neuroprotective effect exerted by pentamidine was confirmed by FJB analysis. A*β* injection caused a significant increase of FJB-positive cell number in CA1 area versus vehicle-treated mice (711 ± 102 versus 101 ± 52, *P* < 0.001 Figures 4(c) and 4(f)). Treatment with pentamidine (0.05–5 *μ*g/mL/day) for 7 days reduced in a concentration-dependent way the number of dying neurons caused by A*β* injection in the same area (352 ± 100, 201 ± 95 and 141 ± 71 versus 101 ± 52, *P* < 0.01 and 0.001 resp., Figures 4(c) and 4(f)).

**Figure 5:**
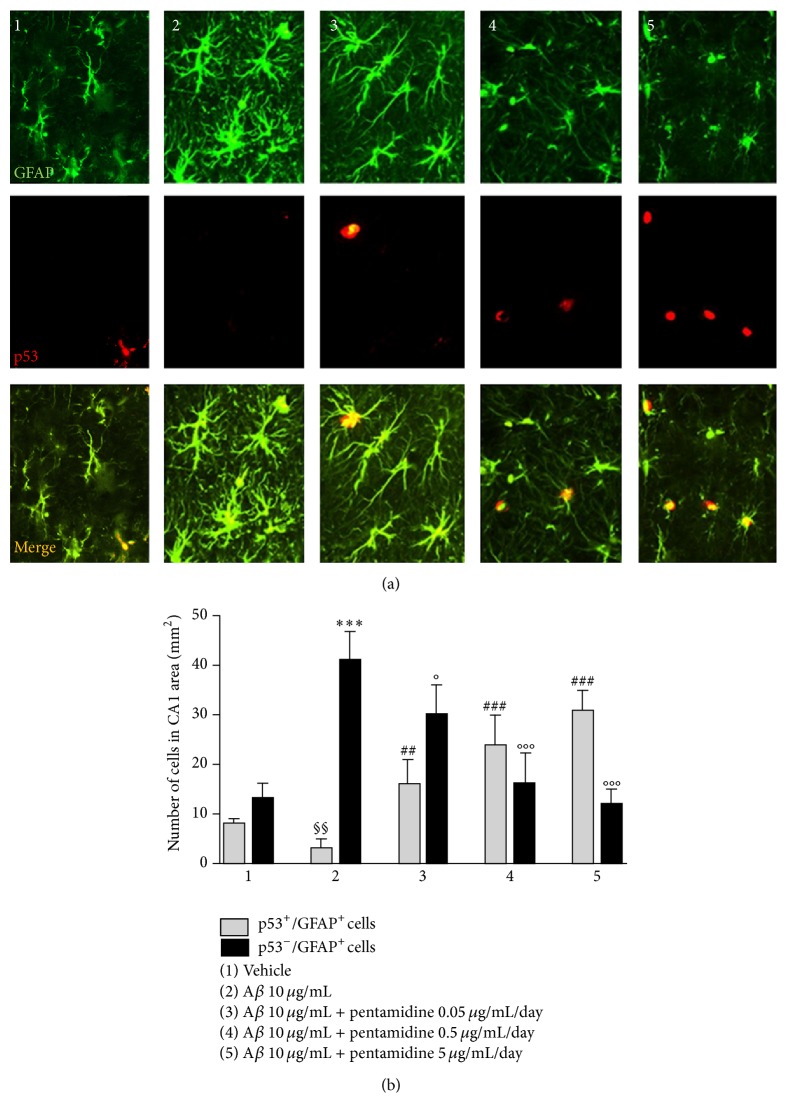
Effect of pentamidine (0.05–5 *μ*g/mL/day) on GFAP and p53 expression in astrocyte in the hippocampi of A*β*-injected mice. (a) Immunofluorescence analysis of hippocampal coronal sections. Note the increased GFAP expression in hippocampal astrocytes of A*β*-treated (2) compared to vehicle-treated mice (1) and the dose-dependent reduction after pentamidine treatment (3-4-5). Scale bar: 50 *μ*m. (b) Relative quantification of p53-positive/GFAP-positive (open bars) and p53-positive/GFAP-positive (filled bars) astrocytes in the CA1 area of the brain. Results are expressed as mean ± SEM of *n* = 4 experiments performed in triplicate. ^*∗∗∗*^ *P* < 0.001 versus vehicle-treated mice; °*P* < 0.05 and °°°*P* < 0.001 versus A*β*-treated mice. ^§§^ *P* < 0.01 versus vehicle-treated mice; ^##^ *P* < 0.01, ^###^ *P* < 0.001 versus A*β*-treated mice.

The neuroprotective effect of pentamidine was accompanied by a significant downregulation of gliosis grade, as shown by the concentration-dependent decrease of GFAP expression (84.6 ± 16, 53 ± 9.6 and 44.2 ± 15, *P* < 0.01 and 0.001, resp.), compared to the hippocampi of A*β*-treated mice (Figures 4(a) and 4(d)). According to the immunohistochemistry, immunofluorescence analysis of GFAP and p53 protein revealed that, after A*β* injection, GFAP+ cell number was significantly increased in the hippocampi of A*β*-compared to vehicle-treated mice (41 ± 6.0 versus 13.0 ± 3.0, *P* < 0.01, Figures 5(a) and 5(b)). Conversely, p53 expression in A*β*-treated mice was significantly reduced compared to vehicle-treated mice (3.2 ± 0.8 versus 9.0 ± 1, *P* < 0.01, Figures 5(a) and 5(b)), very likely as the consequence of astrocytes infiltration. Treatment with pentamidine (0.05–5 *μ*g/mL/day) for 7 days caused a dose-dependent decrease of glial cells as indicated by GFAP positive cell infiltration in CA1 area (30 ± 6.0, 16.0 ± 6.0 and 12.0 ± 3.0 versus 41 ± 6.0, *P* < 0.05 and 0.001, resp., Figures 5(a) and 5(b)) and in the same time it resulted in a dose-dependent increase of nuclear p53 expression in GFAP expressing cells (16.0 ± 5.0, 24.0 ± 6.0 and 31.2 ± 3.0 versus 3.2 ± 0.8, *P* < 0.01 and 0.001 resp., Figures 5(a) and 5(b)).

## 4. Conclusions

Novel therapeutic approaches for the treatment of AD progression should direct towards the (re)discovery of new molecules able to have an impact on several pathological pathways that together converge to the progressive neurological decline characteristic of the disease. Inflammation, more specifically neuroinflammation, has been widely known as an accompanying and key feature in AD [24–26]. In fact, both* in vitro* and* in vivo* studies have shown that A*β*, the major constituent of the senile plaques in the AD brain, can directly or indirectly activate the secretion of proinflammatory cytokines [27]. Therefore, the search for new drugs should be based on diverse targets in the attempt to blunt the inflammatory scenario in the AD brain and not only to replace the neurotransmission failure.

Here we show that pentamidine, an ancient antiprotozoal drug that inhibits S100B protein, ameliorates gliosis and neuroinflammation in a mouse model of A*β*-induced AD. Many studies have been addressed in the attempt to enlarge the pharmacological knowledge on pentamidine and its novel therapeutic effects in disorders characterized by S100B upregulation, such as melanoma [28], glioblastoma [29], and colitis [19]. This has led to the discovery that, besides being an antiprotozoal drug, pentamidine also inhibits S100B activity by blocking the interaction at the Ca^++^/p53 site of the protein. S100B is a unique glial-derived factor in the sense that it is responsible for the establishment of neuroinflammation and neurodegeneration [30]. In fact, in AD brains, S100B is released by reactive astrocytes, a phenomenon known as “reactive gliosis,” and promotes the formation of neurofibrillary tangles in a RAGE-dependent manner [31]. Once released, S100B accumulates at the RAGE [15, 32] and this interaction leads to the induction of lipid peroxidation and to MAPK phosphorylation that in turn converge to NF-*κ*B activation. By triggering this pathway, S100B induces the transcription of proinflammatory proteins and cytokines, such as iNOS protein, IL-1*β*, and TNF*α* [33, 34]. It is thus conceivable that, by specifically targeting the RAGE/S100B interaction in the brain, it would be possible to inhibit S100B-dependent neuroinflammation in AD. Different studies have suggested that a possible therapeutic approach might be the inhibition of the binding of S100B to the V domain of RAGE by using specific antibodies or small molecules [35]. However, since RAGE is not the sole receptor mediating S100B effects, it seems more logic to inhibit the protein itself before it binds to any target. The results of this study demonstrate that pentamidine, via direct inhibition of S100B protein, attenuates 1/reactive gliosis and neuroinflammation induced by A*β* in mouse hippocampi and 2/neuronal loss in the CA1 area of the brain. Specifically, pentamidine caused a dose-dependent decrease of GFAP protein expression, a sign of gliosis, in mice hippocampal homogenates. This was accompanied by the dose-dependent inhibition of iNOS, COX-2, and p-p38 MAPK protein expression. Consequently to S100B inhibition, pentamidine indirectly interferes with S100B-RAGE interaction, leading to a marked inhibition of RAGE protein expression, which was upregulated after A*β* injection. This result caused the interruption of the downstream RAGE-dependent effects such as NF-*κ*B mobilization in the cytosol and the consequent induction of transcription of proinflammatory signaling molecules/cytokines. At confirmation of the amelioration of the inflammatory scenario, pentamidine was also able to reduce the release of proinflammatory cytokines, namely, PGE2 and IL-1*β*. Moreover, we demonstrated that pentamidine inhibited other proinflammatory events like lipid peroxidation and nitric oxide release. According to our previous observations [29], S100B protein release was upregulated by A*β* injection but its level was not affected by pentamidine treatment. To prove that all the above discussed anti-inflammatory effects, together with the reduction of reactive gliosis, were due to the inhibition of S100B/p53 binding, we evaluated p53 expression in the different experimental conditions. We found that the treatment with pentamidine induced p53 expression on infiltrating astrocytes in mouse hippocampi, as sign of enhanced apoptosis. Together with reduced gliosis, we also observed the rescue of neuronal loss in the damaged area of the brain.

Though preliminary, our data identifies in pentamidine a novel potential drug for the treatment of AD features. However, future studies are needed to investigate whether, together with its anti-inflammatory and neuroprotective activity, pentamidine may also improve mnemonic and cognitive performances in experimental models of AD. However, one of the limiting factors of pentamidine resides in its pharmacokinetic profile, which is characterized by low blood brain barrier crossing. Thus, new pharmacokinetic approaches aimed at increasing the delivery of pentamidine into the brain, in combination with a suitable compliance in terms of way of administration, look very intriguing.
